# Supplementary material for: Characterization of QuantiFERON-TB-Plus Results in Patients with Tuberculosis Infection and Multiple Sclerosis
Source: Neurol Int. 2025 Aug 2;17(8):119. doi: 10.3390/neurolint17080119 (PMC12389123; doi:10.3390/neurolint17080119)
Supplement: Supplementary file 1 [file neurolint-17-00119-s001.zip › neurolint-3747334-supplementary.pdf]

biomedicines

MDPI

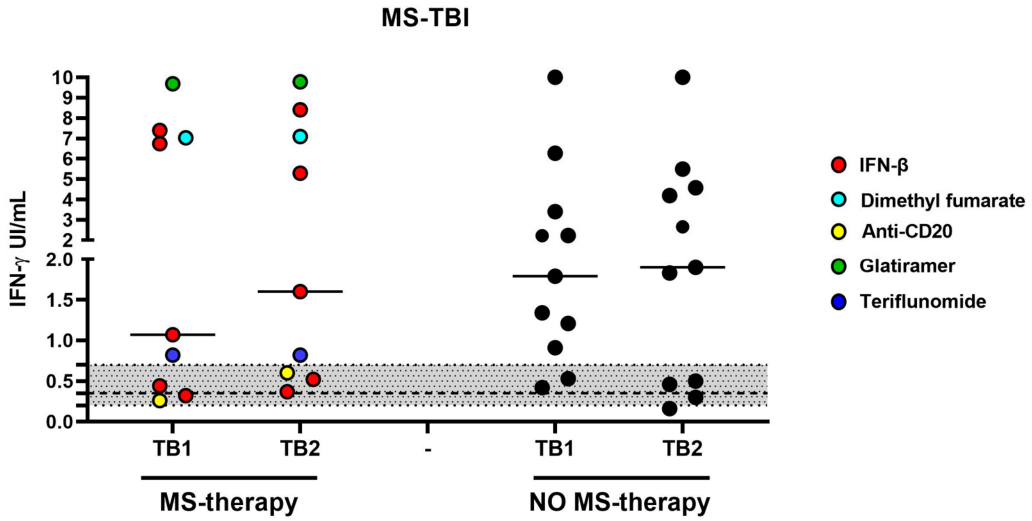

Figure S1. Evaluation of IFN-γ production in response to QFT-Plus antigens in MS-TBI patients stratified according to the MS therapy.
